# Whole-Genome Resequencing to Study Brucellosis Susceptibility in Sheep

**DOI:** 10.3389/fgene.2021.653927

**Published:** 2021-07-08

**Authors:** Xiaolong Li, Qingmin Wu, Xiaoxue Zhang, Chong Li, Deyin Zhang, Guoze Li, Yukun Zhang, Yuan Zhao, Zhaoguo Shi, Weimin Wang, Fadi Li

**Affiliations:** ^1^College of Animal Science and Technology, Gansu Agricultural University, Lanzhou, China; ^2^Engineering Laboratory of Sheep Breeding and Reproduction Biotechnology in Gansu Province, Minqin, China; ^3^The State Key Laboratory of Grassland Agro-ecosystems, College of Pastoral Agriculture Science and Technology, Lanzhou University, Lanzhou, China; ^4^College of Veterinary Medicine, China Agricultural University, Beijing, China

**Keywords:** brucellosis, whole-genome resequencing, *F*_ST_, Fisher’s exact test, chi-square test, sheep

## Abstract

Brucellosis is a zoonotic disease and a major public health problem. However, the genetic mechanism of brucellosis in sheep remains unclear. In this study, serum samples were collected from 6,358 sheep from the F2 population (Dorper sheep ♂ × Hu sheep ♀), and antibody levels were continuously measured at 14 days and 1, 2, 3, 4, 5, 6, 7, 8, 9, and 10 months after administration of brucellosis vaccine. Finally, 19 brucellosis-resistant group (BRG) sheep and 22 brucellosis-susceptible group sheep (BSG) were screened for whole-genome sequencing. Using the fixation index, Fisher’s exact test, and chi-square test, a total of 205 candidate SNP sites were identified. Kyoto Encyclopedia of Genes and Genomes pathway enrichment analysis suggested that 138 candidate genes were significantly enriched in adherens junction (*CTNNA3*, *PARD3*, and *PTPRM*), cell adhesion molecules (*NLGN1*, *CNTNAP2*, *NCAM1*, and *PTPRM*), salivary secretion (*LOC101102109*, *PRKG1*, and *ADCY2*), and hippo signaling pathway (*CTNNA3*, *YAP1*, and *PARD3*). These findings provide valuable molecular markers for brucellosis resistance breeding in sheep and novel insights into the genetic mechanism of brucellosis resistance.

## Introduction

Brucellosis is a common zoonosis (Shakir, 2021). After a brucellosis infection, patients show fever, hyperhidrosis, fatigue, and muscle and joint pain for days or even weeks. Most patients present enlargement of lymph node, liver, spleen, and testicle and other suspicious symptoms and signs that severely affect human health. In addition, it is not easy to differentiate brucellosis from other diseases in the early stage. If not treated in the early stage, there is a high probability that the disease will become chronic and recurrent. Some patients may have the disease for years or even decades. Among animals infected with *Brucella*, female animals often present abortion and infertility, whereas male animals show orchitis, which seriously affects their production performance and hinders the development of animal husbandry, causing serious economic losses. Certain developed countries such as Canada, Britain, the Netherlands, Australia, and other countries have eliminated brucellosis. However, brucellosis continues to pose an economic burden in countries such as Saudi Arabia (Al Jindan, 2021), India (Dhand et al., 2021), and Kenya (Munyua et al., 2021). In conclusion, brucellosis causes huge economic losses in the animal industry and seriously threatens human health (Zhang et al., 2018).

Therefore, it is of great significance to study the genetic mechanism of brucellosis for the development of animal husbandry and human health. *Brucella* infection activates cell-mediated immune responses in animals (Serre et al., 1987). For example, *Brucella abortus* is recognized by several Toll-like receptor-associated pathways, which triggers proinflammatory responses that affect both the type and intensity of the immune response. Toll-like receptor 6 is required to trigger innate immune responses against *B. abortus in vivo* and is required for the full activation of dendritic cells to induce robust proinflammatory cytokine production (de Almeida et al., 2013). Cytokines mediate many of the effector phases of the immune and inflammatory responses. Polymorphisms within the coding and non-coding regions of the cytokine genes may affect the level of cytokine production and regulate the immune response. Polymorphisms of *IL-10* and *TGF-*β*1* genes are associated with susceptibility or resistance to brucellosis. The IL-6 (-174) GC genotype may be a risk factor for the development of focal complications of brucellosis, whereas the GG genotype may be a protective factor against brucellosis (Karaoglan et al., 2009; Amjadi et al., 2019). Therefore, gene polymorphisms can contribute to susceptibility, control, and resistance to treatment in different infectious diseases.

In the present study, we performed whole-genome resequencing of 19 brucellosis-resistant and 22 brucellosis-susceptible sheep. Fixation index (*F*_ST_) has been widely used in the study of candidate genes between groups, including genes associated with traits such as musical aptitude (Liu et al., 2016) and immune function (Mychaleckyj et al., 2017) in humans, vision in sheep (Wang et al., 2019), and immunity and fat deposition in pigs (Qin et al., 2019). Considering the low credibility of using a single method to identify candidate SNP loci, we also used the Fisher test and chi-square test to improve the credibility and accuracy of filtering results. The SNP sites overlapping between these three screening results were selected as candidate sites with significant differences. We aimed to identify genes associated with brucellosis susceptibility in sheep using comparative genomics analysis. Our study could identify valuable molecular markers for brucellosis resistance breeding and providing an important foundation for the study of sheep resistance to brucellosis.

## Materials and Methods

### Ethics Statement

All experiments in this study were carried out in accordance with the approved guidelines from the Regulation of the Standing Committee of Gansu People’s Congress. All experimental protocols, including the sample collection protocol, were approved by the Ethics Committee of Gansu Agriculture University (China) under permission no. DK-005.

### Animals and Blood Serum

A total of 6,358 F2 sheep (Dorper sheep ♂ × Hu sheep ♀) were selected from a sheep farm in Gansu, China. The experimental sheep were all female adult individuals, and there was no historical selection process. Throughout the research process, all the experimental sheep were provided with the same growth environment, the same feeding management, sufficient pellets, and drinking water to ensure that the breeding environment of all sheep was as uniform as possible. The Brucellosis Vaccine (Live, Strain S2) used in this study was purchased from China Qilu Animal Health Co., Ltd. (Jinan, China) (Supplementary Table 1). After lambing, the Brucellosis Vaccine, Live (Strain S2) was diluted to 1.0 ml per sheep, according to the manufacturer’s instructions, and the whole population was subcutaneously inoculated. Blood samples were collected 14 days and 1, 2, 3, 4, 5, 6, 7, 8, 9, and 10 months after vaccination and centrifuged at 7,500 rpm for 5 min; serum was separated, and the antibody status was determined according to the brucellosis Rose Bengal plate agglutination test (RBT) using the method of the Chinese national standard “Diagnostic technology for animal brucellosis GB/T18646–2018.” The standard serum agglutination test (SAT) simultaneously measures the antibody titer and assesses the trend of antibody production and increase in serum levels in sheep after vaccination.

The following procedure was used for the standard SAT procedure for brucellosis in sheep: (1) normal saline containing 0.5% carbolic acid was used to dilute the serum and SAT antigen to be tested. Six test tubes were prepared to dilute each serum sample to be tested; 1.15 ml diluent was added to the first tube, and 0.5 ml diluent was added to the rest of the tubes. (2) Next, 0.1 ml of serum sample was added to the first tube using a 1-ml pipette and mixed well by sucking up the mixture in the test tube into the pipette and then blowing it into the test tube along the wall of the test tube, three to four times. After mixing, 0.25 ml of the mixed solution was sucked up with a pipette and discarded. Then, 0.5 ml of the mixture in the first test tube was added to the second tube using a clean pipette and mixed well using the above mixing. (3) This dilution method was used to complete the dilution in the third, fourth, fifth, and sixth tubes. Finally, 0.25 ml of the mixture in the sixth tube was discarded. After completing the dilution, the dilution degree of the serum of the first to sixth tubes is 1:12.5, 1:25, 1:50, 1:100, 1:200, and 1:400, respectively. (4) Next, 0.5 ml of 20-fold diluted antigen was added to each diluted serum sample and shaken evenly. Thus, the degree of serum dilution was 1:25, 1:50, 1:100, 1:200, 1:400, and 1:800, respectively. Each test tube was incubated at 37°C for 24 h, after which the tubes were checked for self-coagulation. (5) Positive and negative control serum samples and antigen control samples were established for each test. The methods used for dilution and antigen addition for positive and negative control serum samples were the same as described above.

With reference to the turbidity of Wheat-than, the clarity of the supernatant and the degree of bacterial aggregation were determined as follows: (1) Complete bacterial agglutination and 100% precipitation with 100% clarity of the supernatant were judged as “++++.” (2) Almost complete bacterial agglutination and 75% clarity of the supernatant were judged as “+++.” (3) Significant bacterial agglutination and 50% clarity of the supernatant were judged as “++.” (4) Presence of agglutination and precipitation and 25% clarity of the supernatant was judged as “+.” (5) Absence of precipitation of agglomerates and uniformly turbid supernatant was recorded as “−.”

RBT and SAT methods to group experimental sheep. RBT is a qualitative method to determine Brucella positivity and negativity, while SAT is a more accurate and quantitative method to determine Brucella positivity and negativity. RBT and SAT results were divided into positive and negative. Top19 test sheep with negative BRT and the smallest SAT antibody titer were selected as BRG, and Top22 test sheep with positive BRT and the largest SAT antibody titer were selected as BSG.

### DNA Extraction and Resequencing

After antibody detection, jugular vein blood samples (5 ml) from 19 BRG sheep and 22 BSG sheep were collected for DNA extraction. DNA was extracted using an EasyPure Blood Genomic DNA Kit (TransGen Biotech, Beijing, China). DNA was then dissolved in elution buffer (10 mM Tris hydrochloride, 1 mM ethylenediaminetetraacetic acid; pH 8.0) and stored at 20°C. After dilution to 100 ng/μl, the 41 genomic DNA samples were used to generate 41 libraries with a mean insertion size of 500 bp. The libraries were sequenced using 150 bp paired-end reads using an Illumina NovaSeq 6000 system (Illumina, San Diego, CA, United States).

### Sequence Alignment and SNP Calling

FastQC (version 0.10.1) software was used to control the read quality of the original sequence. Linker sequences and sequences containing the uncalled base N were deleted, and the sequences shorter than 60 bp were discarded. The clean reads were mapped onto the sheep reference genome Oar v.4.0 with the Burrows–Wheeler Aligner (BWA, version 0.7.3a) (Li and Durbin, 2009) using the default parameters (bwa mem ref.fna read1.fq read2.fq > aln-pe.sam). Picard (version 1.140) software was used to sort in ascending order the SAM files according to chromosomes and loci to generate BAM files^1^. The duplicates generated because of excessive amplification during library preparation were labeled by Picard software and were not used as evidence for subsequent mutation detection. The Genome Analysis Toolkit (GATK, version 3.4.0) was used to detect variation information in BAM files and generate variant call format (VCF) files. The software VCFtools (version 0.1.14) was used to filter the detection results of SNPs. The screening criteria of SNPs were as follows: (1) the number of support (coverage depth) of SNPs > 3; (2) missing rate per site < 10%; (3) minimum allele frequency (maf) > 5%; (4) removes indel sites. Finally, a total of 6,643,365 SNPs were identified. SNPs were annotated using the ANNOVAR (K. Wang et al., 2010) based on the sheep reference genome Oar v.4.0, and finally the SNPs were divided into exonic regions (variant overlaps a coding), splicing sites (variant is within 2 bp of a splicing junction), intronic regions (variant overlaps an intron), upstream and downstream regions (variant overlap 1-kb region upstream/downstream of transcription start site), and intergenic regions (variant is in the intergenic region) (Supplementary Table 4).

### Statistical Analysis

Three methods, fixation index (*F*_ST_), Fisher’s exact test, and chi-square test, were used to detect SNP sites with significant differences in allele frequency between BRG and BSG. The top 1,000 SNPs were defined as the significantly different loci.

#### Fixation Index

The VCFtools software (version 0.1.14) was used to calculate the fixation index (*F*_ST_ value) of all SNP sites. *F*_ST_ quantifies the allele frequency differences between BRG and BSG, and SNP sites with large *F*_ST_ values may become putative selection sites. The *F*_ST_ values were calculated for each pair of loci following the methods of Weir and Cockerham (Weir and Cockerham, 1984) and using VCFtools (Danecek et al., 2011) from the VCF file. The *F*_ST_ values were arranged in descending order, and the SNPs of the first 1,000 sites were defined as important SNPs. These SNP sites will be used for subsequent candidate gene analysis.

#### Fisher’s Exact Test and Chi-Square Test

Association analysis was performed for BRG group versus BSG group with the PLINK (version 1.9) software, using the Fisher’s exact test and chi-square test (Purcell et al., 2007). Pearson chi-square values and *p*-values (chi-square and Fisher’s exact test) were calculated using Haploview4.2. The first 1,000 SNPs were defined as significant SNPs, which will be used for subsequent analysis of candidate genes.

Finally, to further identify the credible SNPs, the top 1,000 SNPs detected by the above three methods were intersected to identify the overlapping SNPs.

### Gene Annotation and Functional Analysis

The identified SNPs were annotated as the closest genes with a nearest gene distance of 1 kb (Oar_v4.0). The genes were defined as candidate genes. KOBAS software was used to test the statistical enrichment of candidate genes in the Kyoto Encyclopedia of Genes and Genomes (KEGG) pathway analysis (Xie et al., 2011).

## Results

### Brucella Antibody Levels

**FIGURE 1:**
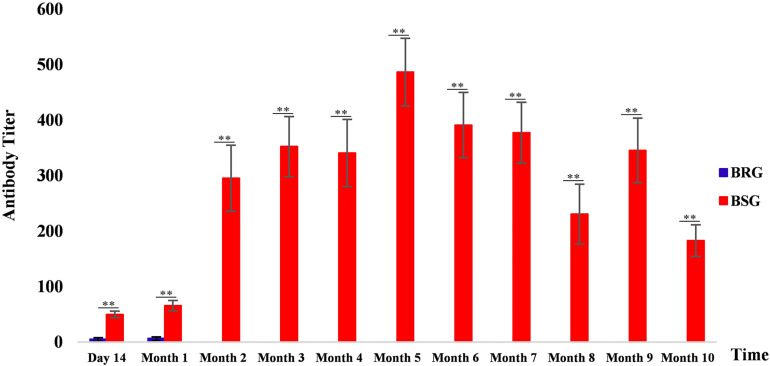
*Brucella* antibody levels in sheep from the brucellosis-resistant group (BRG) and brucellosis-susceptible group (BSG). Blue and red represent the antibody levels of BRG and BSG, respectively, ^∗∗^highly significant difference (Mann–Whitney U test, *p* < 0.01).

Supplementary Table 1 shows the results of antibody titers in the serum agglutination test of 19 BRG and 22 BSG experimental sheep. Figure 1 shows the levels of Brucella antibodies in BRG and BSG. Results indicated that the Brucella antibody titer levels of BSG were significantly higher than those of BRG for 10 consecutive months after vaccination (*p* < 0.01). The results of the agglutination brucellosis Rose Bengal plate agglutination test are shown in Supplementary Figure 1 and Supplementary Table 2.

### Whole-Genome Resequencing and SNP Identification

The Illumina NovaSeq 6000 system was used to sequence the DNA of 41 sheep (BRG, *n* = 19 and BSG, *n* = 22). The system produced a total of 977.167 Gb of raw data. After inspecting the raw data for quality, we filtered out low-quality sequences and sequences containing joints, resulting in 948.21 Gb of clean data. The effective sequencing rate (clean_data/raw_data) was 97.04% (Supplementary Table 2), indicating that the data had high quality and could be used for further in-depth analysis. Using BWA, the clean reads were compared with the Ovis aries reference genome sequence (Oar v4.0), and the average mapped reads and average depth were about 99.75% and 8.5×, respectively (Supplementary Table 3). We filtered out the low-quality SNPs and annotated the detected genetic variations functionally with ANNOVAR software, and 6.64 million SNPs appeared in the final result of annotation (Supplementary Table 4).

### Candidate SNPs and Genes

**FIGURE 2:**
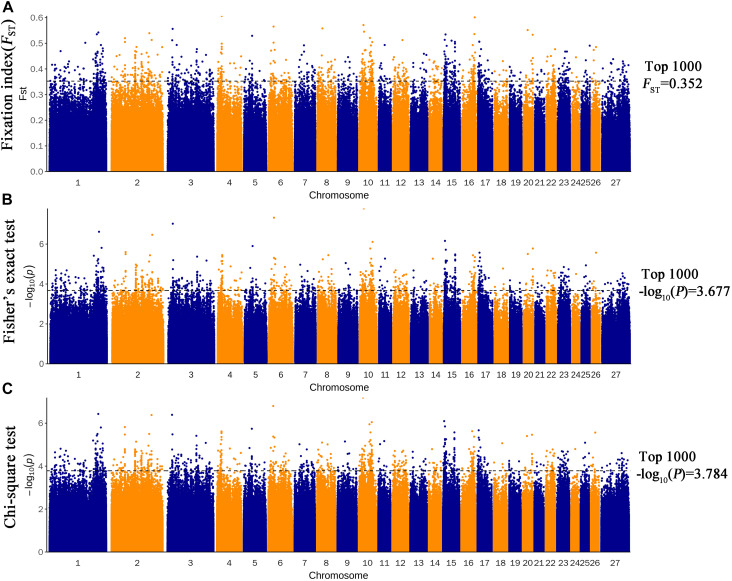
Genome-wide distribution of *F*_ST_ **(A)**, Fisher’s exact test **(B)**, and chi-square test **(C)**. The horizontal black line in the figure shows the values for the first 1,000.

**FIGURE 3:**
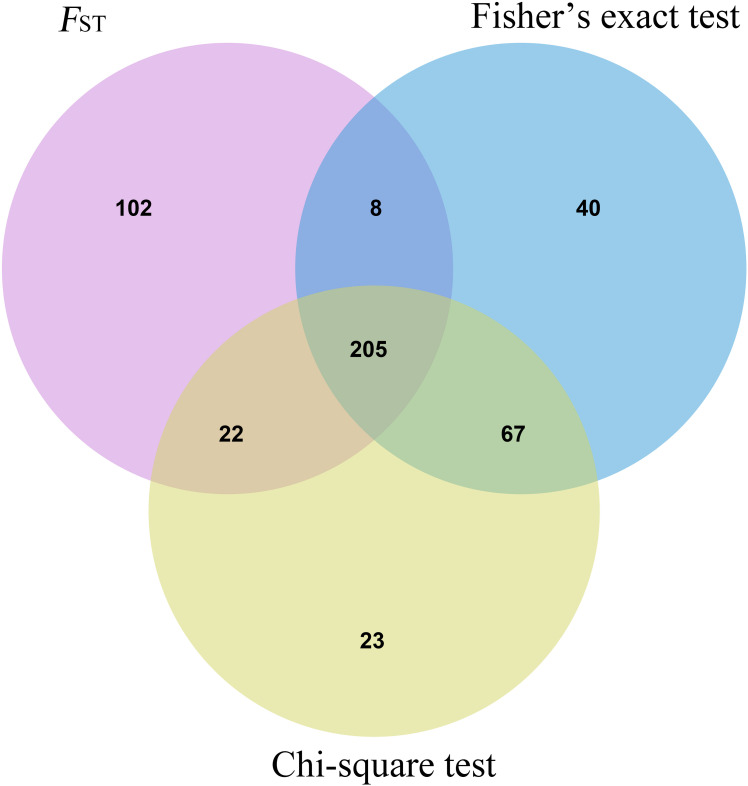
Shared SNP sites Venn diagram of three statistical methods.

Three methods were used to identify SNPs and genes associated with Brucella susceptibility in sheep. Manhattan plots of genome-wide *F*_ST_, Fisher’s exact test, and chi-square test analyses are depicted in Figure 2. First, we extracted the top 1,000 SNPs by *F*_ST_ value (Supplementary Table 5) and –log10(P) value of Fisher’s exact test (Supplementary Table 6) and chi-square test (Supplementary Table 7), respectively. The top 1,000 SNPs were annotated to the closest gene (Oar_v4.0), and these genes were defined as candidate genes. The list of candidate genes and SNPs identified by the three methods is as follows: *F*_ST_: 229 candidate genes and 337 SNPs (Supplementary Table 8), Fisher’s exact test: 193 candidate genes and 320 SNPs (Supplementary Table 9), and chi-square test: 192 candidate genes and 317 SNPs (Supplementary Table 10). Intersection of the three groups of identified genes and SNPs showed that only a small fraction of SNPs could be detected by only one of the three methods, whereas most SNPs overlapped between the three methods; 138 candidate genes and 205 overlapping SNPs were identified (Figure 3 and Supplementary Table 11).

### KEGG Enrichment Analysis

**FIGURE 4:**
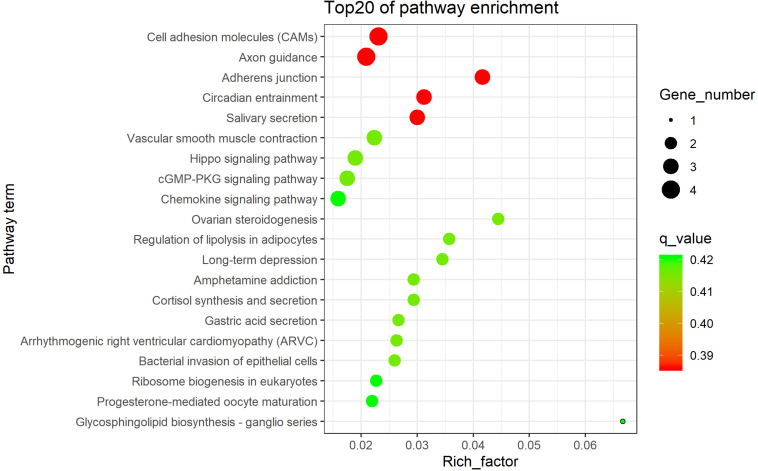
The top 20 KEGG enrichment pathways.

The 138 overlapped genes obtained above were analyzed using KEGG pathway enrichment analysis; the top 20 enriched pathways are shown in Figure 4. Subsequently, four significantly enriched KEGG pathways (*p* < 0.05) were identified: adherens junction, cell adhesion molecules (CAMs), salivary secretion, and Hippo signaling pathway (Table 1 and Supplementary Table 12).

**TABLE 1:** Four significantly enriched KEGG pathways.

Related pathways	*p*-value	Related genes
Adherens junction	0.00669	*CTNNA3*, *PARD3*, *PTPRM*
Cell adhesion molecules (CAMs)	0.01296	*NLGN1*, *CNTNAP2*, *NCAM1*, *PTPRM*
Salivary secretion	0.01582	*LOC101102109*, *PRKG1*, *ADCY2*
Hippo signaling pathway	0.04946	*CTNNA3*, *YAP1*, *PARD3*

## Discussion

In this study, three methods [fixation index (*F*_ST_), Fisher’s exact test, and chi-square test] were used to identify Brucella susceptibility genes in the sheep genome. The experimental sheep were grouped on the basis of the serum levels of Brucella antibodies of different individuals. Sheep with low serum antibody persistence were classified as brucellosis susceptible, and those with high serum antibody persistence were classified as brucellosis resistant. Then, SNP loci showing differences in allele frequency between BRG and BSG were identified as candidate loci closely associated with Brucella resistance (Figure 2).

In the end, 205 overlapping SNPs annotated 138 genes, and some of these genes contain multiple SNP sites (Supplementary Table 11); this finding is likely because genes associated with disease phenotypes often contain multiple SNP loci. Genes known to be highly associated with brucellosis contain multiple SNP sites, such as *TGF-β* (Bravo et al., 2008; Rafiei et al., 2007), *IFN-γ* (Bravo et al., 2003; Rasouli and Kiany, 2007; Hedayatizadeh-Omran et al., 2010; Eskandari-Nasab et al., 2013), *TNF-α* (Caballero et al., 2000; Davoudi et al., 2006; Batikhan et al., 2010), IL-15 (Kalani et al., 2011), and *TNF-α* (Caballero et al., 2000; Kalani et al., 2011).

The 138 genes identified above were used for KEGG pathway enrichment analysis to explore the putative biological functions of these genes. The KEGG pathway analysis identified a total of 12 significant KEGG pathways (Supplementary Table 12), and we mainly focused on the pathways closely related to Brucella infection, which were mainly associated with cell adhesion (Table 1). Brucella invasion is the first step in which phagocytes play a role; the bacteria first adhere to host cell membrane through lipopolysaccharide receptors and lipid valves. FC receptors play a role in processes such as intrusion of bacteria into the cell to reproduce, transfer through blood, and lymph node invasion of host organs through adhesion, host response, organ inflammation, and organ lesion (Amjadi et al., 2019), such as membranoproliferative glomerulonephritis (Provatopoulou et al., 2018), testicular abscess (Vallianou et al., 2018), and splenic abscess (Sudulagunta et al., 2017).

In pathways associated with adherens junctions and CAMs, genes closely related to cell adhesion—such as *CTNNA3*, *PARD3*, *PTPRM*, *NLGN1*, *CNTNAP2*, and *NCAM1*—play a role. CTNNA3 mediates cell adhesion (Janssens et al., 2001); when *CTNNA3* was knocked down, CTNNA3-depleted cells showed abnormal skeleton (Stahn et al., 2016). In esophageal cancer, *PARD3* overexpression promotes cell apoptosis, inhibits cell proliferation, and inhibits cell migration and invasion, whereas *PARD3* silencing promotes cell proliferation and increases migration and invasion (Wang et al., 2017). *PTPRM* overexpression in small intestinal neuroendocrine tumor cell lines reduced cell growth and proliferation and induced apoptosis (Barazeghi et al., 2019). Neuroligins are cell differentiation molecules located on the postsynaptic side of the synapse, which interact with their presynaptic partners neurexins to maintain trans-synaptic connection (Südhof, 2008). Mutations in the *CNTNAP2* gene are partly responsible for autism and other disorders as the gene regulates neural connections in the frontal lobes (Scott-Van Zeeland et al., 2010). TGF-β1 regulates the adhesion properties of cardiomyocytes through an NCAM1-dependent mechanism and is detrimental to the heart (Ackermann et al., 2017). These genes may be related to the transfer of Brucella in the body and associated pathogenic processes, but these findings need further verification to confirm their role in the pathogenesis of brucellosis.

Adherence of Brucella to human epithelial cells and macrophages is mediated by sialic acid residues (Castañeda-Roldán et al., 2004). In our study, salivary secretion was identified as a significant KEGG pathway; *PRKG1* and *ADCY2* are involved in cell adhesion in different diseases. For example, *PKAG1* mutations drive the relaxation of aortic smooth muscle cells and induce aortic disease (Milewicz et al., 2017), Brucella can cause recurrent episodes of vascular inflammation (Korkmaz et al., 2016). ADCY2 can catalyze the formation of signal molecule cAMP in response to G protein signal transduction, and downstream signal transduction cascade mediates the change in gene expression patterns, leading to increased IL-6 production (Ding et al., 2004), which is involved in the development of the chief complications of brucellosis (Karaoglan et al., 2009).

Functional deletions in the Hippo signaling pathway indicate significant overgrowth in the Hippo signaling pathway owing to increased cell proliferation and reduced cell death (Pan, 2010). The complexity of regulation of YAP1 and TAZ was greatly increased, and more regulatory components were discovered. The Hippo pathway is inter-linked with other cancer-related pathways, especially transforming growth factor-β (TGF-β) and WNT pathways (Moroishi et al., 2015). *TGF-β1* may be involved in susceptibility to brucellosis and development of main forms of the disease (Bravo et al., 2008). It is speculated that these genes play an important role in the pathogenesis of brucellosis, but further studies are needed to confirm these findings.

Unfortunately, no SNP loci reached significant levels after *p*-value correction using FDR and Bonferroni methods (corrected *p-*value < 0.05) in this study, which may be due to the small sample size. Therefore, in the follow-up study, the sample size should be expanded and the FDR or Bonferroni methods should be used for screen candidate genes and SNP sites.

## Conclusion

In conclusion, four significant pathways and nine candidate genes related to brucellosis susceptibility in sheep were identified by whole-genome resequencing using brucellosis-susceptible and brucellosis-resistant sheep. These pathways and genes are closely related to the process of cell adhesion. These results provide valuable candidate genes and lay an important foundation for the study of brucellosis in sheep.

## Data Availability Statement

The datasets presented in this study can be found in online repositories. The names of the repository/repositories and accession number(s) can be found below: https://www.ncbi.nlm.nih.gov/, SRR11971264–SRR11971304.

## Ethics Statement

The animal study was reviewed and approved by Ethics Committee of Gansu Agriculture University (China). Written informed consent was obtained from the owners for the participation of their animals in this study.

## Author Contributions

FL and WW designed the experiments. XL and WW analyzed the data. XL wrote the manuscript. CL, DZ, GL, XZ, YKZ, YZ, and ZS contributed to sample collection and prepared biological samples. WW, QW, and XL revised the manuscript. All authors read and approved the final manuscript.

## Conflict of Interest

The authors declare that the research was conducted in the absence of any commercial or financial relationships that could be construed as a potential conflict of interest.

## Footnotes

1 http://broadinstitute.github.io/picard

## References

[B1] Ackermann M. A. Petrosino J. M. Manring H. R. Wright P. Shettigar V. Kilic A. (2017). TGF-β1 affects cell-cell adhesion in the heart in an NCAM1-dependent mechanism. *J. Mol. Cell. Cardiol.* 112 49–57. 10.1016/j.yjmcc.2017.08.015 28870505 PMC5647243

[B2] Al Jindan R. (2021). Scenario of pathogenesis and socioeconomic burden of human brucellosis in Saudi Arabia. *Saudi J. Biol. Sci.* 28 272–279. 10.1016/j.sjbs.2020.09.059 33424306 PMC7783673

[B3] Amjadi O. Rafiei A. Mardani M. Zafari P. Zarifian A. (2019). A review of the immunopathogenesis of brucellosis. *Infect. Dis.* 51 321–333. 10.1080/23744235.2019.1568545 30773082

[B4] Barazeghi E. Hellman P. Westin G. Stålberg P. (2019). PTPRM, a candidate tumor suppressor gene in small intestinal neuroendocrine tumors. *Endocr. Connect.* 8 1126–1135. 10.1530/EC-19-0279 31349215 PMC6687034

[B5] Batikhan H. Gokcan M. K. Beder E. Akar N. Ozturk A. Gerceker M. (2010). Association of the tumor necrosis factor-alpha -308 G/A polymorphism with nasal polyposis. *Eur. Arch. Otorhinolaryngol.* 267 903–908. 10.1007/s00405-009-1167-5 20012441

[B6] Bravo M. J. Colmenero J. D. Queipo-Ortuño M. I. Alonso A. Caballero A. (2008). TGF-beta1 and IL-6 gene polymorphism in Spanish brucellosis patients. *Cytokine* 44 18–21. 10.1016/j.cyto.2008.07.008 18804384

[B7] Bravo M. J. de Dios Colmenero J. Alonso A. Caballero A. (2003). Polymorphisms of the interferon gamma and interleukin 10 genes in human brucellosis. *Eur. J. Immunogenet.* 30 433–435. 10.1111/j.1365-2370.2003.00419.x 14675398

[B8] Caballero A. Bravo M. J. Nieto A. Colmenero J. D. Alonso A. Martín J. (2000). TNFA promoter polymorphism and susceptibility to brucellosis. *Clin. Exp. Immunol.* 121 480–483. 10.1046/j.1365-2249.2000.01331.x 10971514 PMC1905734

[B9] Castañeda-Roldán E. I. Avelino-Flores F. Dall’Agnol M. Freer E. Cedillo L. Dornand J. (2004). Adherence of brucella to human epithelial cells and macrophages is mediated by sialic acid residues. *Cell. Microbiol.* 6 435–445. 10.1111/j.1462-5822.2004.00372.x 15056214

[B10] Danecek P. Auton A. Abecasis G. Albers C. A. Banks E. DePristo M. A. (2011). The variant call format and VCFtools. *Bioinformatics* 27 2156–2158. 10.1093/bioinformatics/btr330 21653522 PMC3137218

[B11] Davoudi S. Amirzargar A. A. Hajiabdolbaghi M. Rasoolinejad M. Soodbakhsh A. Jafari S. (2006). Th-1 cytokines gene polymorphism in human brucellosis. *Int. J. Immunogenet.* 33 355–359. 10.1111/j.1744-313x.2006.00626.x 16984280

[B12] de Almeida L. A. Macedo G. C. Marinho F. A. V. Gomes M. T. R. Corsetti P. P. Silva A. M. (2013). Toll-like receptor 6 plays an important role in host innate resistance to *Brucella abortus* infection in mice. *Infect. Immun.* 81 1654–1662. 10.1128/IAI.01356-12 23460520 PMC3647997

[B13] Dhand N. K. Singh J. Josan H. S. Singh B. B. Jaswal N. Tiwari H. K. (2021). The feasibility and acceptability of various bovine brucellosis control strategies in India. *Prev. Vet. Med.* 189:105291. 10.1016/j.prevetmed.2021.105291 33582551

[B14] Ding Q. Gros R. Gray I. D. Taussig R. Ferguson S. S. G. Feldman R. D. (2004). Raf kinase activation of adenylyl cyclases: isoform-selective regulation. *Mol. Pharmacol.* 66 921–928. 10.1124/mol.66.4.921 15385642

[B15] Eskandari-Nasab E. Moghadampour M. Hasani S.-S. Hadadi-fishani M. Mirghanizadeh-Bafghi S.-A. Asadi-Saghandi A. (2013). Relationship between γ-interferon gene polymorphisms and susceptibility to brucellosis infection. *Microbiol. Immunol.* 57 785–791. 10.1111/1348-0421.12093 24033468

[B16] Hedayatizadeh-Omran A. Rafiei A. Hajilooi M. Haghshenas M. (2010). Interferon-gamma low producer genotype +5644 over presented in patients with focal brucellosis. *Pak. J. Biol. Sci.* 13 1036–1041. 10.3923/pjbs.2010.1036.1041 21313874

[B17] Janssens B. Goossens S. Staes K. Gilbert B. van Hengel J. Colpaert C. (2001). Alphat-catenin: a novel tissue-specific beta-catenin-binding protein mediating strong cell-cell adhesion. *J. Cell Sci.* 114(Pt 17) 3177–3188. 10.1242/jcs.114.17.3177 11590244

[B18] Kalani M. Rasouli M. Moravej A. Kiany S. Rahimi H. R. (2011). Association of interleukin-15 single nucleotide polymorphisms with resistance to brucellosis among Iranian patients. *Tissue Antigens* 78 352–358. 10.1111/j.1399-0039.2011.01775.x 21988722

[B19] Karaoglan I. Pehlivan S. Namiduru M. Pehlivan M. Kilinçarslan C. Balkan Y. (2009). TNF-alpha, TGF-beta, IL-10, IL-6 and IFN-gamma gene polymorphisms as risk factors for brucellosis. *New Microbiol.* 32 173–178. 19579695

[B20] Korkmaz P. Kıdır M. Namdar N. D. Özmen A. Uyar C. Değer A. N. (2016). A case of brucellosis with recurrent attacks of vasculitis. *Case Rep. Infect. Dis.* 2016:5740589. 10.1155/2016/5740589 27042369 PMC4794566

[B21] Li H. Durbin R. (2009). Fast and accurate short read alignment with burrows-wheeler transform. *Bioinformatics* 25 1754–1760. 10.1093/bioinformatics/btp324 19451168 PMC2705234

[B22] Liu X. Kanduri C. Oikkonen J. Karma K. Raijas P. Ukkola-Vuoti L. (2016). Detecting signatures of positive selection associated with musical aptitude in the human genome. *Sci. Rep.* 6:21198. 10.1038/srep21198 26879527 PMC4754774

[B23] Milewicz D. M. Trybus K. M. Guo D.-C. Sweeney H. L. Regalado E. Kamm K. (2017). Altered smooth muscle cell force generation as a driver of thoracic aortic aneurysms and dissections. *Arterioscler. Thromb. Vasc. Biol.* 37 26–34. 10.1161/ATVBAHA.116.303229 27879251 PMC5222685

[B24] Moroishi T. Hansen C. G. Guan K.-L. (2015). The emerging roles of YAP and TAZ in cancer. *Nat. Rev. Cancer* 15 73–79. 10.1038/nrc3876 25592648 PMC4562315

[B25] Munyua P. Osoro E. Hunsperger E. Ngere I. Muturi M. Mwatondo A. (2021). High incidence of human brucellosis in a rural Pastoralist community in Kenya, 2015. *PLoS Negl. Trop. Dis.* 15:e0009049. 10.1371/journal.pntd.0009049 33524052 PMC7877737

[B26] Mychaleckyj J. C. Havt A. Nayak U. Pinkerton R. Farber E. Concannon P. (2017). Genome-wide analysis in Brazilians reveals highly differentiated native American genome regions. *Mol. Biol. Evol.* 34 559–574. 10.1093/molbev/msw249 28100790 PMC5430616

[B27] Pan D. (2010). The hippo signaling pathway in development and cancer. *Dev. Cell* 19 491–505. 10.1016/j.devcel.2010.09.011 20951342 PMC3124840

[B28] Provatopoulou S. Papasotiriou M. Papachristou E. Gakiopoulou H. Marangos M. Goumenos D. S. (2018). Membranoproliferative glomerulonephritis in a patient with chronic brucellosis. *Kidney Res. Clin. Pract.* 37 298–303. 10.23876/j.krcp.2018.37.3.298 30254855 PMC6147194

[B29] Purcell S. Neale B. Todd-Brown K. Thomas L. Ferreira M. A. R. Bender D. (2007). PLINK: a tool set for whole-genome association and population-based linkage analyses. *Am. J. Hum. Genet.* 81 559–575. 10.1086/519795 17701901 PMC1950838

[B30] Qin M. Li C. Li Z. Chen W. Zeng Y. (2019). Genetic diversities and differentially selected regions between shandong indigenous pig breeds and western pig breeds. *Front. Genet.* 10:1351. 10.3389/fgene.2019.01351 32038711 PMC6987402

[B31] Rafiei A. Hajilooi M. Shakib R. J. Alavi S. A. (2007). Transforming growth factor-beta1 polymorphisms in patients with brucellosis: an association between codon 10 and 25 polymorphisms and brucellosis. *Clin. Microbiol. Infect.* 13 97–100. 10.1111/j.1469-0691.2006.01575.x 17184296

[B32] Rasouli M. Kiany S. (2007). Association of interferon-gamma and interleukin-4 gene polymorphisms with susceptibility to brucellosis in Iranian patients. *Cytokine* 38 49–53. 10.1016/j.cyto.2007.05.003 17566759

[B33] Scott-Van Zeeland A. A. Abrahams B. S. Alvarez-Retuerto A. I. Sonnenblick L. I. Rudie J. D. Ghahremani D. (2010). Altered functional connectivity in frontal lobe circuits is associated with variation in the autism risk gene CNTNAP2. *Sci. Transl. Med.* 2:56ra80. 10.1126/scitranslmed.3001344 21048216 PMC3065863

[B34] Serre A. Bascoul S. Vendrell J. P. Cannat A. (1987). Human immune response to Brucella infection. *Ann. Inst. Pasteur Microbiol.* 138 113–117. 10.1016/0769-2609(87)90088-3 3300715

[B35] Shakir R. (2021). Brucellosis. *J. Neurol. Sci.* 420:117280. 10.1016/j.jns.2020.117280 33358192

[B36] Stahn V. Nagel I. Fischer-Huchzermeyer S. Oyen F. Schneppenheim R. Gesk S. (2016). Molecular analysis of hybrid neurofibroma/schwannoma identifies common monosomy 22 and α-T-Catenin/CTNNA3 as a novel candidate tumor suppressor. *Am. J. Pathol.* 186 3285–3296. 10.1016/j.ajpath.2016.08.019 27765635

[B37] Südhof T. C. (2008). Neuroligins and neurexins link synaptic function to cognitive disease. *Nature* 455 903–911. 10.1038/nature07456 18923512 PMC2673233

[B38] Sudulagunta S. R. Kumbhat M. Sodalagunta M. B. Settikere Nataraju A. Bangalore Raja S. K. (2017). Isolated splenic abscess in brucellosis. *Oxf. Med. Case Rep.* 2017:omx001. 10.1093/omcr/omx001 28473914 PMC5410879

[B39] Vallianou N. G. Melaki K. Constantinou F. Gennimata V. Kokkinakis E. (2018). Testicular abscesses due to *Brucella melitensis*. *New Microbes New Infect.* 26 1–2. 10.1016/j.nmni.2018.08.010 30245825 PMC6141671

[B40] Wang K. Li M. Hakonarson H. (2010). ANNOVAR: functional annotation of genetic variants from high-throughput sequencing data. *Nucleic Acids Res.* 38:e164. 10.1093/nar/gkq603 20601685 PMC2938201

[B41] Wang L. Zhang H. Hasim A. Tuerhong A. Hou Z. Abdurahmam A. (2017). Partition-Defective 3 (PARD3) regulates proliferation, apoptosis, migration, and invasion in esophageal squamous cell carcinoma cells. *Med. Sci. Monit.* 23 2382–2390. 10.12659/msm.903380 28526815 PMC5446977

[B42] Wang W. Zhang X. Zhou X. Zhang Y. La Y. Zhang Y. (2019). Deep genome resequencing reveals artificial and natural selection for visual deterioration, plateau adaptability and high prolificacy in Chinese domestic sheep. *Front. Genet.* 10:300. 10.3389/fgene.2019.00300 31001329 PMC6454055

[B43] Weir B. S. Cockerham C. C. (1984). Estimating F-statistics for the analysis of population structure. *Evol. Int. J. Org. Evol.* 38 1358–1370. 10.1111/j.1558-5646.1984.tb05657.x 28563791

[B44] Xie C. Mao X. Huang J. Ding Y. Wu J. Dong S. (2011). KOBAS 2.0: a web server for annotation and identification of enriched pathways and diseases. *Nucleic Acids Res.* 39 W316–W322. 10.1093/nar/gkr483 21715386 PMC3125809

[B45] Zhang N. Huang D. Wu W. Liu J. Liang F. Zhou B. (2018). Animal brucellosis control or eradication programs worldwide: a systematic review of experiences and lessons learned. *Prev. Vet. Med.* 160 105–115. 10.1016/j.prevetmed.2018.10.002 30388992

