# Supplementary material for: Whole-Genome Resequencing to Study Brucellosis Susceptibility in Sheep
Source: Front Genet. 2021 Jul 8;12:653927. doi: 10.3389/fgene.2021.653927 (PMC8297390; doi:10.3389/fgene.2021.653927)
Supplement: Supplementary file 2 [file Data_Sheet_2.docx]

**Whole-genome resequencing to study brucellosis susceptibility in sheep**

Xiaolong Li^1^, Qingmin Wu^4^, Xiaoxue Zhang ^1,2^, Chong Li ^1,2^, Deyin Zhang ^1^, Guoze Li ^1^, Yukun Zhang ^1^, Yuan Zhao ^1^, Zhaoguo Shi ^1^, Weimin Wang ^1^, Fadi Li ^1,2,3^

**Supplementary Figures**


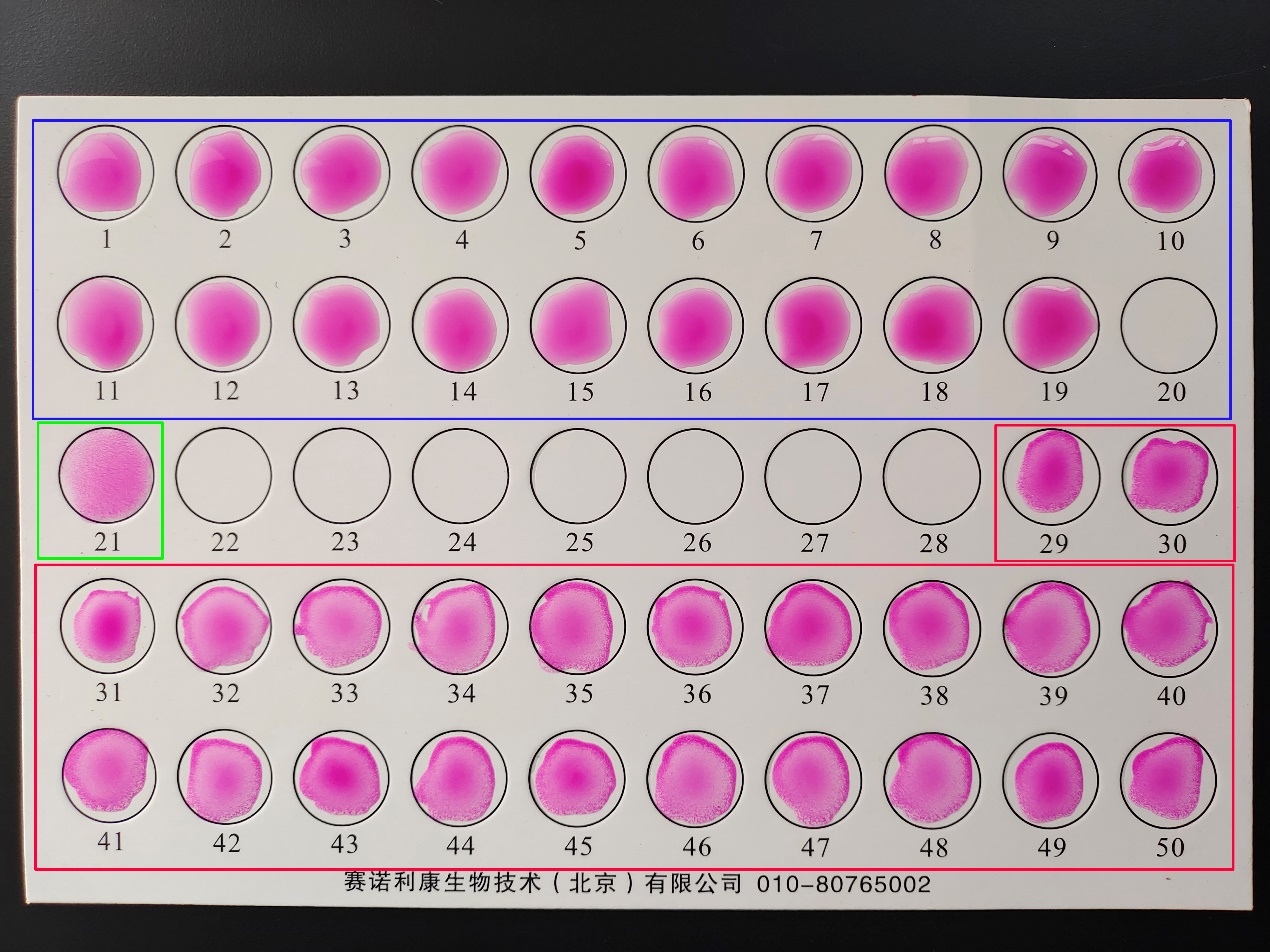


**Figure. S1** Brucellosis rose-bengal plate agglutination test of BRG and BSG. The blue wire frame represents BRG, the red wire frame represents BSG, and the green wire frame represents the positive control.
